# Alirocumab Attenuated Plaque Inflammation and PCSK9-Induced Proinflammatory Signalling in M1 Macrophages Independently of Lipid Lowering

**DOI:** 10.3390/biom16030397

**Published:** 2026-03-06

**Authors:** Cristina Espadas, Manuel Soto-Catalán, María Romero-Cote, María Kavanagh, Isabel Herrero-Del Real, Adriana Ortega-Hernández, Jairo Lumpuy-Castillo, Dulcenombre Gómez-Garre, Jesús Egido, José Tuñón, Carmen Gómez-Guerrero, Óscar Lorenzo

**Affiliations:** 1 Laboratory of Vascular Pathology and Diabetes, Fundación Instituto de Investigaciones Sanitarias-Fundación Jiménez Díaz, Universidad Autónoma, 28040 Madrid, Spain; cristina.espadas@estudiante.uam.es (C.E.); manuel.sotoc@estudiante.uam.es (M.S.-C.); maria.rcote.edu@quironsalud.es (M.R.-C.); maria.kavanagh@estudiante.uam.es (M.K.); isabelm.herrero@estudiante.uam.es (I.H.-D.R.); jairo.lumpuy@estudiante.uam.es (J.L.-C.); jegido@quironsalud.es (J.E.); cgomezg@fjd.es (C.G.-G.); 2 Spanish Biomedical Research Centre in Diabetes and Associated Metabolic Disorders (CIBERDEM) Network, 28029 Madrid, Spain; 3 Laboratory of Vascular Biology and Microbiota, Instituto de Investigaciones Sanitarias Hospital Clínico San Carlos, 28040 Madrid, Spain; adriana.ortega@salud.madrid.org (A.O.-H.); mgomezgarre@salud.madrid.org (D.G.-G.); 4 Department of Cardiology, Hospital Fundación Jiménez Díaz, Universidad Autónoma, 28040 Madrid, Spain; jtunon@quironsalud.es; 5 Spanish Biomedical Research Centre in Cardiovascular Diseases (CIBERCV) Network, 28029 Madrid, Spain

**Keywords:** PCSK9, alirocumab, inflammation, NFκB, NLRP3

## Abstract

Background: Proprotein Convertase Subtilisin/Kexin Type 9 (PCSK9) has been implicated in vascular inflammation beyond its action on LDL-C degradation. We investigated whether PCSK9 may exacerbate proinflammatory signaling of M1 macrophages and if its neutralization with alirocumab could attenuate this effect and plaque progression by LDL-C independent mechanisms. Methods: ApoE^−^/^−^ mice were treated with alirocumab for 13 weeks, and aortic arches were isolated for atherosclerotic plaque characterization based on lesion size and lipid and macrophage infiltration. Plasma and splenic monocytes/macrophages were also assessed by flow cytometry, and PCSK9, the lipid profile, and inflammatory cytokines were measured by qPCR or Western blot. Cultured THP-1-derived M1 macrophages were stimulated with PCSK9 and evaluated for TLR4-NFκB-NLRP3 activation and cytokine production. In addition, soluble PCSK9, LDL-C, and proinflammatory factors were analyzed in 1190 patients with acute coronary syndrome (ACS). Results: Alirocumab reduced plaque lesion (0.42-fold; *p* < 0.05) and lipid (0.63-fold; *p* < 0.01) and macrophage (0.61-fold; *p* < 0.05) infiltration, mainly the M1 subtype (0.37-fold; *p* < 0.01), as well as TLR4, NLRP3 and caspase-1 expressions (0.49-fold, 0.51-fold and 0.51-fold, respectively; *p* < 0.05), without altering LDL-C. Also, it decreased proinflammatory cytokines but enhanced anti-inflammatory factors and M2 markers at the descending aorta. Alirocumab enriched circulating Ly6C^low^ monocytes (1.51-fold; *p* < 0.05) and splenic M2 macrophages (1.32-fold; *p* < 0.01), while reducing M1 (0.62-fold; *p* < 0.05). In cultured M1 macrophages, PCSK9 overexpressed proinflammatory cytokines (i.e., CXCL9, CXCL10, TNF-α, IL-1β, and IL-6), downregulated anti-inflammatory mediators (i.e., CCL17, TGM2, TGF-β1, and IL-10), and promoted NFκB-p65 nuclear translocation and NLRP3 and gasdermin-D activation. However, TLR4 inhibition or silencing blunted these effects. In patients with AC, there was a positive association between PCSK9 and hsCRP and FGF-23 plasma levels, independently of LDL-C. Conclusions: PCSK9 may be released in parallel to proinflammatory factors such as hsCRP and FGF-23 in patients with ACS, independently of LDL-C levels. PCSK9 may directly promote macrophage-driven inflammatory responses through the TLR4-NFκB-NLRP3 signaling, but its neutralization with alirocumab attenuated this inflammatory axis and limited atherosclerotic progression, supporting an anti-inflammatory benefit secondary to PCSK9 inhibition.

## 1. Introduction

Atherosclerosis is a chronic inflammatory disease initiated and sustained by the accumulation of lipids, particularly oxidized low-density lipoproteins (LDL) within the artery wall, which triggers complex inflammatory responses involving vascular and immune cells [1]. Particularly, monocytes drive atherogenesis by infiltrating into atherosclerotic plaques, differentiating into macrophages, and, upon lipid uptake, becoming foam cells that amplify inflammation and plaque growth. Moreover, macrophages can polarize to proinflammatory M1 or tissue-repair M2 macrophages in response to multiple signals from the microenvironment [2]. Activation of nuclear factor-κB (NFκB), primarily through Toll-like receptors (TLR), can be a crucial factor promoting the M1 phenotype (suppressing M2 polarization) to overexpress proinflammatory cytokines, intensifying endothelial activation and monocyte recruitment [3]. Also, activation of the NOD-like receptor-3 (NLRP3)-inflammasome pathway can lead to the upregulation of M1-associated cytokines, such as IL-1β and CXCL10, and a decrease in M2-associated factors like ArgI and CD206 [4]. Thus, although lipid accumulation remains a key driver of plaque formation, the persistent inflammatory milieu may further increase residual cardiovascular risk [5]. In this sense, new anti-atherosclerotic therapies could address vascular inflammation to protect from cardiovascular injuries.

Traditional anti-atherosclerosis drugs, particularly statins, possess hypolipidemic and anti-inflammatory properties [6]. However, vascular inflammation may persist despite effective lipid control, indicating the involvement of lipid-independent regulatory mechanisms. In this context, the pro-protein convertase subtilisin/kexin type 9 (PCSK9) reduces the LDL receptor (LDLR)-mediated clearance of atherogenic lipoproteins such as LDL-C [7]. PCSK9 has also been implicated in the upregulation and recruitment of macrophages, thereby contributing to localized inflammatory processes within vascular tissues [8]. In fact, PCSK9 inhibitors such as alirocumab reduced plasma LDL-C and plaque progression [9], but their potential direct macrophage-driven anti-inflammatory actions have not been completely elucidated. Previous studies have shown that PCSK9 gene silencing directly lessened macrophage infiltration and cytokine level via the TLR4-NFκB pathway at the atherosclerotic plaque of ApoE^−^/^−^ mice [10]. However, whether PCSK9 influences the maintenance and amplification of established macrophage inflammatory phenotypes and downstream NLRP3 activation has not been fully explored. Thus, we aim to investigate whether PCSK9 may act as an amplifier of M1 macrophage polarization and local and systemic inflammation, and whether its pharmacological neutralization with alirocumab interferes with local and systemic inflammatory pathways via TLR4-NFκB and NLRP3.

## 2. Methods

### 2.1. Human Study

To investigate the potential association between PCSK9 and systemic inflammation, we quantified circulating PCSK9 levels alongside a panel of inflammatory biomarkers in a cohort of 1190 patients with acute coronary syndrome (ACS) with/without electrocardiographic ST-segment elevation of the BACS-BAMI (Biomarkers in ACS and Biomarkers in Acute Myocardial Infarction) study carried out in five hospitals in Madrid. The research protocol included patients admitted to the hospitals Fundación Jiménez Díaz, Fuenlabrada, Móstoles, and Alcorcón in Madrid with either non-ST elevation acute coronary syndrome (NSTEACS) or ST elevation myocardial infarction (STEMI). NSTEACS was defined as angina at rest lasting >20 min in the previous 24 h, or new-onset class III to IV angina, along with transient ST depression or T-wave inversion on the electrocardiogram considered diagnostic by the attending cardiologist, and/or troponin elevation. STEMI was defined as symptoms compatible with angina lasting >20 min, ST elevation in 2 adjacent leads on the electrocardiogram without response to nitroglycerine, and troponin elevation. Exclusion criteria were age > 85 years, coexistence of other significant cardiac disorders except left ventricular hypertrophy secondary to hypertension, coexistence of any illness or toxic habits that could limit patient survival, impossibility to perform revascularization when indicated, subjects in whom follow-up was not possible, and those who were not clinically stable after six days from ACS [11]. On admission, clinical variables were recorded, and 12 h fasting blood was taken for analysis. Then, EDTA-plasma samples were immediately isolated by blood centrifugation at 2500 rpm (20 min) at 4 °C and stored at −80 °C until use. PCSK9 was determined using the enzyme-linked immunosorbent assay (ELISA) with an anti-PCSK9-specific antibody (ELLA kit SPCKB-PS00321, ProteinSimple, Bio-Techne, Minneapolis, MN, USA). LDL-C concentrations were determined using standardized enzymatic methods on the ADVIA 2400 Chemistry System (Siemens Healthineers, Munich, Germany). Also, a specific panel of proinflammatory factors composed of high-sensitivity C-reactive protein (hs-CRP), monocyte chemoattractant protein-1 (MCP-1), fibroblast growth factor-23 (FGF-23), and interleukin-18 (IL-18) was quantified using the latex-enhanced immunoturbidimetry (ADVIA 2400 Chemistry System), the ELISA kits (DCP00 human MCP-1 from R&D Systems, Minneapolis, MN, USA, and human FGF23-C-Term from Immutopics Inc., San Clemente, CA, USA), and the ELLA kit SPCKB-PS000501 for IL-18.

### 2.2. In Vivo Studies

#### 2.2.1. Mouse Model of Atherosclerosis

Apolipoprotein E-deficient (ApoE^−^/^−^) mice (B6.129P2-Apoetm1Unc/J; The Jackson Laboratory, Bar Harbor, ME, USA, stock no. 002052, RRID:IMSR_JAX:002052), congenic on C57BL/6J after >10 backcross generations, were used as a model of hypercholesterolemia and accelerated atherosclerosis due to impaired ApoB- and ApoE-rich lipoprotein clearance, resulting in inefficient cholesterol removal and elevated plasma LDL-cholesterol (LDL-C) levels [12]. The ApoE^−^/^−^ mouse model provides a stringent platform to interrogate LDL-C–independent immunomodulatory effects of PCSK9 neutralization, as PCSK9 blockade does not significantly reduce circulating cholesterol in the absence of functional ApoE/LDLR-dependent lipoprotein clearance [13]. We fed ApoE^−^/^−^ mice (males, 10–12 weeks old; *n* = 16) a Western diet (42% fat, 0.21% cholesterol; ref: D12079, Ssniff Spezialdiäten, Soest, Germany) for 15 weeks to accelerate plaque progression and complexity, providing an experimental model that better resembles advanced human disease [14]. Animals were housed in groups of 4–5 per ventilated cage (20–22 °C) and maintained on a 12 h light/dark cycle, with ad libitum access to food and water. At the 2nd week of the Western diet, animals were randomly assigned to a control group (*n* = 9) or to a treatment group (*n* = 7) with the PCSK9 monoclonal antibody, alirocumab (10 mg/kg/week divided into two s.c. injections). Our timeline and dosing strategy were selected based on established preclinical regimens [9,15,16] and were specifically intended to maintain sustained PCSK9 blockade during advanced plaque inflammation rather than to model clinical LDL-C responses. A minimum of seven animals per group was selected using variance estimates from published studies and assuming α = 0.05 and β = 0.2.

Then, at the 15th week, animals were euthanized under anesthesia (ketamine, 100 mg/kg, and xylazine, 10 mg/kg) administered via intraperitoneal injection, and total peripheral blood, aortic arch, the descending aorta, spleen, and liver were collected. All procedures were approved by the FISS-FJD Animal Experimentation Ethics Committee and the Regional Government (Ref. PROEX 128.4-23) on 6 June 2023 in accordance with EU Directive 2010/63/EU and national regulations (RD 53/2013).

#### 2.2.2. Anthropometric, Biochemical, and Cytokine Measurements

The animals’ body weight was recorded weekly. None of the mice showed infectious events along the model, and no differences were observed in the food and water intake. Plasma was obtained by centrifuge-separation (20 min, 2500 rpm, 4 °C) from peripheral blood in EDTA-containing tubes. The lipid profile was assessed using specific commercial kits for total cholesterol (TC) (Cell Biolabs, San Diego, CA, USA, #STA-390), triglycerides (TG) (Cayman Chemical, Ann Arbor, MI, USA, #10010303), and LDL-C (CrystalChem, Downers Grove, IL, USA, #79980). Plasma PCSK9 was evaluated by ELISA assay (R&D Systems, Minneapolis, MN, USA, #MPC-900), whereas TNF-α and IL-6 were quantified using the Simple Plex™ ELLA automated immunoassay system (ProteinSimple, Bio-Techne). Moreover, liver PCSK9 expression was studied by Western blot using anti-PCSK9 (D7U6L) rabbit monoclonal primary antibody (Cell Signaling, Danvers, MA, USA, #85813), as described below.

#### 2.2.3. Quantification of Atherosclerotic Lesions and Macrophage Infiltration

During sacrifice, mice were perfused from the heart with 10 mL of cold PBS, and the entire aorta was collected. The aortic arch was embedded in OCT compound (Sigma-Aldrich, St. Louis, MO, USA, #6502) and stored at –80 °C. The aortic area of atherosclerotic plaque (μm^2^) was quantified from 1000 μm of the cardiac valve leaflets in serial sections of 7 μm obtained by cryostat (Leica CM1520, Biosystems, Wetzlar, Germany). Sections were fixed with cold acetone (10 min, −20 °C), washed (60% isopropanol), and stained for one hour with 2.5 mg/mL oil-red-O (ORO) (Sigma Aldrich, St. Louis, MO, USA, #O1391) and hematoxylin. The major lesion of atherosclerotic plaque was established in those sections with the maximal stained area. This region was quantified as the ratio of lesion area to the total aortic surface under microscopy (Zeiss Axioscope 5, Carl Zeiss Microscopy GmbH, Oberkochen, Germany). In addition, the neutral lipid content was also quantified by ORO/hematoxylin within these sections.

Total macrophage infiltration at the atheroma plaques was evaluated by incubating OCT cryosections (just adjacent to the maximal atherosclerotic lesion) with an anti-CD68 antibody (Abcam, Cambridge, UK, #125212). First, sections were fixed in cold acetone, and endogenous peroxidase was quenched with methanol. Non-specific protein binding was blocked with goat serum (1 h, room temperature), and primary anti-CD68 antibody (1:400) was incubated overnight at 4 °C, followed by a biotinylated anti-rabbit secondary antibody (1:500, 1 h, room temperature, Themo Fisher Scientific, Waltham, MA, USA, #11859200). The macrophage staining was developed by using the avidin–biotin complex (Vector Laboratories, Newark, NJ, USA, #PK-7100), revealed with 3,3′-diaminobenzidine (DAB) (Abcam, #ab64238), and counterstained with hematoxylin. In all cases, for each mouse, two sections and four fields per section were analyzed with ImageJ software (v. 1.54).

#### 2.2.4. Characterization of Macrophage Infiltration in the Aortic Arch

Infiltrated macrophages were then characterized for M1 phenotype. Cryosections were fixed with 4% paraformaldehyde (PFA) for 10 min at room temperature, followed by blockade of non-specific binding using goat serum. Samples were then incubated overnight with anti-CD80 primary antibody (1:50, 4 °C, Abcam, #ab254579), followed by a goat anti-rabbit IgG Alexa Fluor 488-conjugated secondary antibody (1:300, 1 h, room temperature, Themo Fisher Scientific, #A-11008). Nuclear counterstaining was performed using DAPI for 10 min. After washing, sections were mounted, and images were acquired using a Leica TCS SP5 confocal microscope (10× objective, 2.5× digital zoom, Leica Biosystems, Wetzlar, Germany). Fluorescence quantification was performed using ImageJ software (v. 1.54).

Also, expressions of TLR4, caspase-1 and NLRP3 were analyzed in these atherosclerotic lesions enriched in M1 macrophages (CD68^+^/CD80^+^) by immunohistochemistry with anti-TLR4 (Abcam, #218987), -caspase-1 (Santa Cruz, Dallas, TX, USA, #sc-514) and -NLRP3 (Affinity-Bionova, Cincinnati, OH, USA, #DF15549), respectively, at 1:100 dilution, followed by a biotinylated anti-rabbit secondary antibody (1:200, 1 h, room temperature). Protein staining was revealed with the avidin–biotin complex and DAB, as described above.

#### 2.2.5. Aortic Gene Expression

Descending aorta was disaggregated using 1 mm diameter zirconium beads and a homogenizer (Bullet Blender Homogenizer, Next Advance Inc., Troy, NY, USA). Total RNA was isolated using TRIzol reagent (ThermoFisher Scientific, #15596026; Waltham, MA, USA) according to the manufacturer’s protocol. RNA concentration and purity were assessed spectrophotometrically (Nanophotometer^®^ N60, IMPLEN, Munich, Germany). The cDNA synthesis was performed using the High-Capacity cDNA Reverse Transcription Kit (#4368813) on a Veriti Thermal Cycler (ThermoFisher Scientific). Quantitative real-time PCR (qPCR) was carried out in 10 µL reactions containing cDNA, TaqMan™ Universal PCR Master Mix (ThermoFisher Scientific, #4318157), and gene-specific TaqMan assays *CXCL10* (Mm00445235_m1), *TNF-α* (Mm00443258_m1), *IL-1β* (Mm00434228_m1), *MCP-1* (Mm00441242_m1), *IL-6* (Mm00446190_m1), *TGF-β1* (Mm01178820_m1), *IL-10* (Mm00439614_m1), *CD163* (Mm00474091_m1), *ArgII* (Mm00477592_m1), and *CD206* (Mm01329359_m1)] on a StepOnePlus™ Real-Time PCR System (ThermoFisher Scientific). All samples were run in triplicate, and those with cycle threshold (C_T_) variation exceeding 0.3 were excluded. The relative gene expression was calculated using the comparative ∆∆C_T_ method, with *β-actin* (Mm02619580_g1) serving as the endogenous control.

#### 2.2.6. Flow Cytometry Analysis

Characterization of monocyte and macrophage populations was achieved in peripheral blood and spleen. First, 200 µL of whole EDTA-blood was aliquoted and stabilized with Transfix Bulk (CytoMark, Buckingham, UK, 300 K Solutions, #TFB-20-1), according to the manufacturer’s instructions. Specific antibodies against monocyte markers (see below) were added to the samples and incubated for 30 min at 4 °C. Then, the BD lysis buffer (BD Bioscience, San Jose, CA, USA, #349202) was added for 10 min, and samples were vortexed to lyse red blood cells, which were removed after centrifugation. The pellet with stained cells was washed and resuspended in cold PBS. On the other hand, spleens were harvested and kept in cold PBS to be mechanically disaggregated and filtered through 70 µm and 40 µm microfilters (ThermoFisher Scientific, #10788201). Similarly, cell lysates were incubated with specific antibodies and fixed with 1% PFA in PBS.

For both blood and spleen samples, we tested a comprehensive panel of fluorochrome-conjugated antibodies targeting key monocyte and macrophage markers (BioLegend, San Diego, CA, USA). We used: APC/Cyanine7 anti-mouse CD45 (clone 30-F11, #103116), APC anti-mouse/human CD11b (clone M1/70, #101212), FITC anti-mouse lymphocyte antigen 6 complex at locus C (Ly6C, clone HK1.4, #128006), PE/Cyanine7 anti-mouse F4-80 recombinant antibody (clone QA17A29, #157308), PE anti-Nos2 (iNOS) (clone W16030C, #696806), and Brilliant Violet 421™ anti-mouse CD206 (MMR) (clone C068C2, #141717). Moreover, CD45^+^/CD11b^+^-monocytes were further classified by the presence of Ly6C. Those Ly6C^high^ monocytes express higher levels of Ly6C and are mostly involved in proinflammatory activities, whereas Ly6C^low^ (lower Ly6C) corresponds to monocytes involved in tissue repair and anti-inflammation [17]. Similarly, CD45^+^/F4-80^+^-macrophages were classified according to their polarization state as proinflammatory (M1) or anti-inflammatory (M2) macrophages. M1 macrophages were identified by the expression of inducible nitric oxide synthase (iNOS), while M2 macrophages were defined by the expression of the macrophage mannose receptor, CD206 [18]. Then, flow cytometric acquisition was performed on a Cytoflex (Beckman Coulter, Brea, CA, USA), acquiring 10,000 live events from peripheral blood and 50,000 live events from spleen suspensions. Data analysis was conducted using CytExpert 2.3 and Kaluza Analysis Software v. 2.4 (Beckman Coulter).

### 2.3. In Vitro Studies

#### 2.3.1. Monocytes Culture, Differentiation, and Polarization

The human THP-1 cell line of monocytes was donated by Dr. Martín-Ventura and cultured in the Roswell Park Memorial Institute medium (RPMI 1640) supplemented with glutamine 2.05 mM (Gibco, Waltham, MA, USA, #61870036), 10% heat-inactivated fetal bovine serum (Sigma-Aldrich, #F7524), and 1% Penicillin–Streptomycin (Sigma-Aldrich, #P0781), and maintained at 37 °C and 5% CO_2_ in a humidified incubator. Monocytes were counted, seeded (1.5 × 10^6^ cells/p60-plate), and differentiated into naïve macrophages (M0) by adding 100 nM PMA (phorbol 12-myristate 13-acetate, Sigma-Aldrich, #P8139) for 72 h. Differentiated macrophages were washed and primed for 48 h with M1-polarization medium composed of 20 ng/mL IFN-γ (PeProtech, Rocky Hill, CT, USA, #300-02) and 10 pg/mL LPS (*Escherichia coli* O111:B4, Sigma-Aldrich) dissolved in RPMI-1640 (0.5% FBS) [19,20]. M1 macrophages were confirmed by assessing the increased cytokine CXCL9/CCL17 ratio [21,22].

#### 2.3.2. Stimulation of M1 Macrophages

M1 macrophages were stimulated for 3–24 h with 2.5 ug/mL human recombinant PCSK9 (hPCSK9, Sigma-Aldrich, #SRP6285) in M1-polarization medium, as previously described [8]. This concentration was considered a physiologically relevant upper bound for mechanistic in vitro assays [23]. hPCSK9 was certified as endotoxin-free, excluding potential confounding effects of lipopolysaccharide contamination. TNF-α (100 ng/mL; PeProtech, #300-01A) or LPS (1 ug/mL; Sigma-Aldrich, #L3012) was used as a positive control of pro-inflammation. Some cells were also pre-treated with parthenolide (1 h, 10 µM; Sigma-Aldrich) as an NFκB inhibitor preventing IκBα degradation, or with MCC950 (10 µM; InvivoGen, San Diego, CA, USA) as a selective NLRP3 inhibitor that blocks its oligomerization and caspase-1 activation. TAK-242 (10 µM; MedChemExpress, Monmouth Junction, NJ, USA) and alirocumab (1μM) were used as specific TLR4 and PCSK9 inhibitors, respectively. All compounds except alirocumab were dissolved in DMSO, and vehicle-treated cells served as controls.

#### 2.3.3. TLR4-Gene Silencing

Small interfering (si) RNA sequence for the human TLR4 gene was purchased from ThermoFisher Scientific (Assay ID: S14195, #4390824). M1 macrophages were transfected with siRNA-TLR4 dissolved in Lipofectamine^®^ RNAiMAX reagent following the manufacturer’s instructions (Invitrogen, ThermoFisher #13778100). After 24 h, cells were washed with PBS and stimulated with PCSK9 or LPS. Confirmation of gene and protein silencing was done by qPCR and Western blot (Supplementary Figure S1A).

#### 2.3.4. Macrophages’ Gene and Protein Expression

Total RNA was isolated from cultured M1 macrophages and converted to cDNA for quantification of relative gene expression by qPCR, as described above. The human taqman assay IDs were as follows: *CXCL9* (Hs00171065_m1), *CXCL10* (Hs00171042_m1), *IL-1β* (Hs01555410_m1), *TNF-α* (Hs00174128_m1), *IL-6* (Hs0174131_m1), *CCL17* (Hs00171074_m1), *TGM2* (Hs01096681_m1), *IL-10* (Hs00961622_m1), *TGF-β1* (Hs00998133_m1), and *TLR4* (Hs00152939_m1), with *18S* rRNA (Hs99999901_s1) serving as the endogenous control.

Total proteins were also extracted from M1 macrophages by adding lysis buffer (Tris 50 mM, NaCl 0.15 M, EDTA 2.5 mM, TRITON X-100 0.2%, and IGEPAL 0.3%). Protein concentration was determined with the Pierce™ BCA Protein Assay Kit (ThermoFisher Scientific, #23225). Then, 20 ug per sample were loaded on SDS-PAGE gels, separated by electrophoresis, and transferred onto PVDF membranes (ThermoFisher Scientific, #88520). Non-specific binding was blocked with 5% skimmed milk in Tris Buffer Saline-Tween 20, and primary antibodies were used to quantify the protein levels: anti-Phospho-IκBα (Ser32) rabbit monoclonal (Cell Signaling), -IκB-αlpha (L35A5) mouse monoclonal (Cell Signaling), -Phospho-NFκB p65 (Ser536) rabbit monoclonal (Cell Signaling), -RELA/NFκB p65 (F-6) (Santa Cruz), -NLRP3 rabbit monoclonal (Cell Signaling), -Cleaved gasdermin-D rabbit monoclonal (Cell Signaling), and -TLR4 (Abcam, #218987), with -GAPDH (Invitrogen) or -β-actin (Sigma-Aldrich) antibodies serving as endogenous control. Secondary antibodies were goat anti-Mouse IgG (H + L) and goat anti-Rabbit IgG (H + L) (ThermoFisher Scientific, #G21040 and #G21234, respectively). The antibody–antigen binding was detected by chemiluminescence (iBright750, Invitrogen, Carlsbad, CA, USA) and quantified by the Quantity One 4.6.6 software (BioRad, Hercules, CA, USA). All experiments were done at least three times.

#### 2.3.5. Cell Immunofluorescence

Nuclear translocation of NFκB-p65 was detected by immunofluorescence. THP-1 cells (6.0 × 10^4^ cells/well) were seeded in 8-well chamber slides, differentiated, and polarized to M1, as described above. Cells were stimulated with PCSK9 for 30 min, washed, and fixed with 4% PFA in PBS (10 min, 4 °C), followed by blocking (1% BSA) and permeabilization (0.1% Triton X-100, 1 h, room temperature). Then, cells were incubated with primary anti-p65 (SantaCruz, Dallas, TX, USA, #sc-8008) (1:80) at 4 °C overnight, and secondary anti-mouse AlexaFluor-488 conjugated secondary antibody (1:200 dilution, 1 h, ThermoFisher, #A-11001). The cell nuclei were stained with DAPI, and images were obtained by a confocal microscope (Leica TCS SP5). Quantification was performed using ImageJ, counting 30 cells per field within five different fields. All experiments were done at least three times.

### 2.4. Statistical Analysis

Data distribution in the human study was assessed using the Kolmogorov–Smirnov test to evaluate normality. Variables with non-normal distribution were expressed as median (interquartile range) and compared using the Kruskal–Wallis test, followed by Dunn’s post hoc correction for multiple comparisons. A quantile regression model was also applied to explore the relationship between plasma PCSK9 levels and selected clinical variables at 25th, 50th, and 75th percentiles (τ = 0.25, 0.50, 0.75) of the outcome distribution. For descriptive purposes and graphical visualization, hsCRP, FGF-23, and LDL-C were categorized into tertiles. The tertiles refer to groups defined according to the distribution of hsCRP, FGF23, and LDL-C within the ACS cohort, using two cut-off points corresponding to the 33.3rd and 66.7th percentiles (T1–T3). Then, PCSK9 concentrations were compared across these tertiles using non-parametric Kruskal–Wallis tests. When overall differences were identified, pairwise comparisons between tertiles were performed using Dunn’s post hoc test. Two-tailed *p*-values < 0.05 were considered statistically significant.

Data from animals and cells are expressed as mean ± standard deviation (SD) unless otherwise indicated. Normality was assessed using the Shapiro–Wilk test. Comparisons between two groups were performed using an unpaired two-tailed Student’s *t*-test. For comparisons involving more than two groups, one-way analysis of variance (ANOVA), followed by Dunnett or Tukey’s post hoc test, was used. Non-parametric data were analyzed using Mann–Whitney *U* or Kruskal–Wallis tests, as appropriate. A *p*-value < 0.05 was considered statistically significant. Statistical analyses were performed using GraphPad Prism version 8.0.1 (GraphPad Software, San Diego, CA, USA).

## 3. Results

### 3.1. Association of PCSK9 and Proinflammatory Biomarkers in Acute Coronary Syndrome

**Figure 1:**
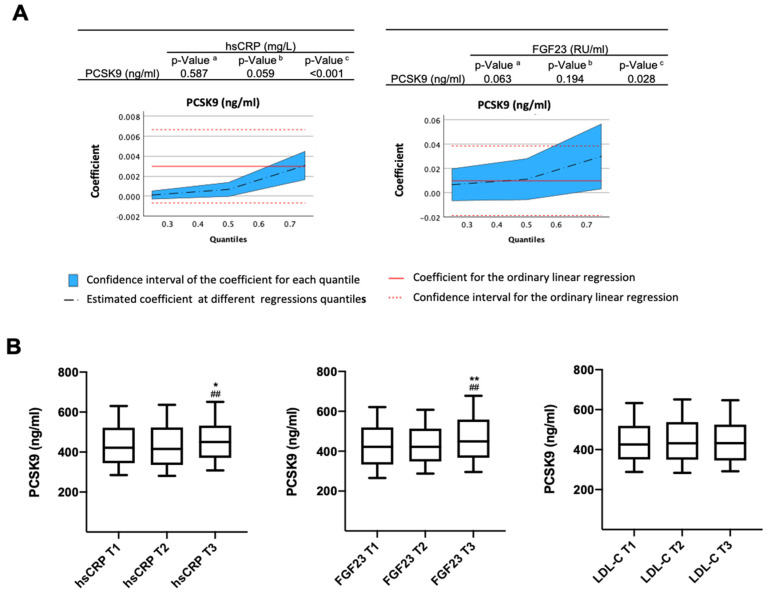
Plasma PCSK9 and proinflammatory factors after acute coronary syndrome. (**A**) Associations between PCSK9 and hsCRP and FGF23 plasma levels. Coefficients were estimated at the 0.25 (a), 0.50 (b), and 0.75 (c) quantiles of each factor by using quantile regression models adjusted for sex, age, BMI, LDL-C, previous statin or ezetimibe use, coronary artery disease, hypertension, type 2 diabetes, dyslipidemia, and LDL-C. Additional adjustment for hsCRP was done when analyzing the association between PCSK9 and FGF23. A *p* < 0.05 was considered significant. (**B**) For descriptive purposes and graphical visualization, hsCRP, FGF-23, and LDL-C were categorized into tertiles, and the distribution of PCSK9 was analyzed. Data are shown as box plots, including median and 25th–75th percentiles (whiskers represent the 10th–90th percentiles). * *p* < 0.05 and ** *p* < 0.01 vs. T1; ^##^ *p* < 0.01 vs. T2.

We analyzed the potential association between PCSK9 and proinflammatory cytokines, including hsCRP, MCP-1, FGF-23, and IL-18, in plasma from 1190 patients with ACS [11]. Briefly, subjects were 61.6 years old, 77.1% of them were men, they showed a body mass index of 28.3 kg/m^2^, and they exhibited hypertension (57.1%), dyslipemia (60.1%), and previous coronary artery disease (20.2%). By quantile regression, we observed a significant association of PCSK9 with hsCRP and FGF-23 at the 0.75 quantile (75th percentile), even after adjustment for sex, age, BMI, and additionally, LDL-C, previous statin or ezetimibe use, and previous coronary artery disease, hypertension, dyslipidemia, or type 2 diabetes. The PCSK9 association with FGF23 was additionally adjusted by hsCRP (Figure 1A). In fact, PCSK9 concentrations were significantly elevated in the third tertile (T3) relative to the first and second tertiles (T1 and T2) of both hsCRP and FGF-23, whereas no association was observed with LDL-C levels (Figure 1B). These findings indicate that, in patients with ACS, elevated PCSK9 levels are associated with increased circulating concentrations of hsCRP and FGF-23, but not with LDL-C, thereby supporting a lipid-independent proinflammatory role of PCSK9 in this condition.

### 3.2. Alirocumab Reduced Inflammation but Not Hyperlipidemia in ApoE^−^/^−^ Mice

**Figure 2:**
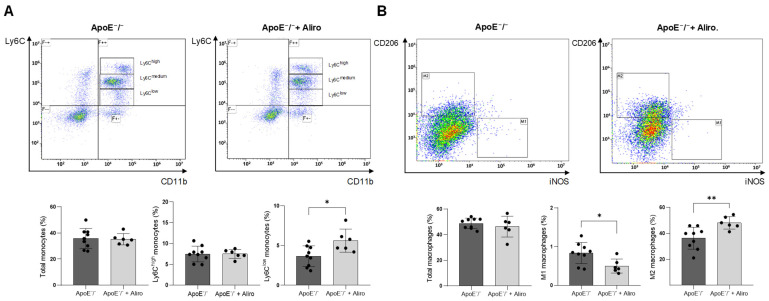
Alirocumab enhanced Ly6C^low^ monocytes and M2 macrophages in ApoE^−^/^−^ mice. (**A**) Top, gating analysis for CD11b and Ly6C (high, medium, and low phenotypes) positive staining in plasma monocytes from ApoE^−^/^−^ and ApoE^−^/^−^ + alirocumab mice. Bottom, quantification of total, Ly6C^high^, and Ly6C^low^ monocytes. (**B**) Top, gating analysis for iNOS and CD206 positive staining in spleen macrophages from ApoE^−^/^−^ and ApoE^−^/^−^ + alirocumab mice. Bottom, quantification of total, M1, and M2 macrophages. * *p* < 0.05 and ** *p* < 0.01 vs. ApoE^−^/^−^ mice, by unpaired two-tailed Student’s *t*-test. *N* = 6–9, per group.

As previously described [24], after 15 weeks of the Western diet, ApoE^−^/^−^ mice showed higher levels of plasma lipids (total cholesterol, LDL-cholesterol, and triglycerides) and increased levels of proinflammatory cytokines TNF-α and IL-6 (Table 1). Administration of the PCSK9-neutralizing antibody, alirocumab (10 mg/kg/week), for 13 weeks overexpressed liver and plasma PCSK9 (Supplementary Figure S1B), as observed in humans [24], but did not modify the body weight and lipid profile (Table 1). However, alirocumab significantly attenuated serum levels of TNF-α and IL-6 (Table 1) and reduced the proinflammatory phenotype of circulating mononuclear cells. By flow cytometry, we observed an augmented number of anti-inflammatory CD45^+^/CD11^+^-Ly6C^low^ monocytes in alirocumab-treated mice compared to untreated animals (5.6 ± 1.5% vs. 3.6 ± 1.3%, respectively; *p* < 0.05), while the proinflammatory CD45^+^/CD11^+^-Ly6C^high^ monocytes were not altered (Figure 2A). In the spleen, alirocumab also reduced the number of proinflammatory M1 (CD45^+^/F4-80^+^/iNOS^+^) macrophages (0.5 ± 0.2% vs. 0.8 ± 0.3%, respectively; *p* < 0.05) and enhanced the M2 (CD45^+^/F4-80^+^/CD206^+^) macrophages (48.0 ± 4.5% vs. 36.3 ± 8.5%, respectively; *p* < 0.01) (Figure 2B).

### 3.3. Alirocumab Reduced Plaque Lesions and Local Inflammation in ApoE^−^/^−^ Mice

**Figure 3:**
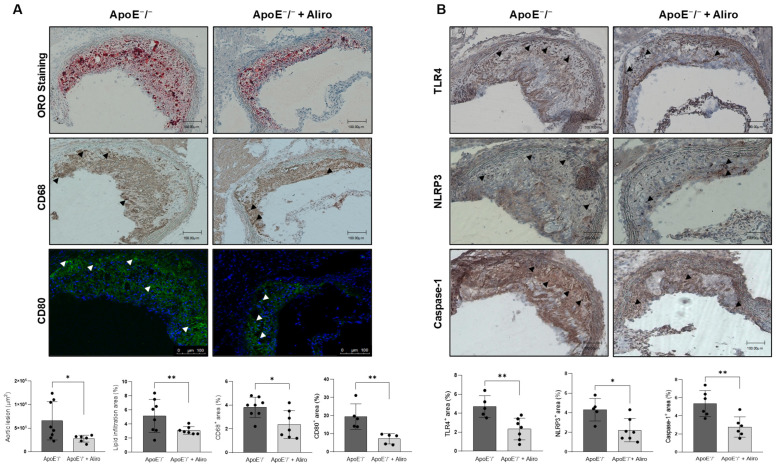
Alirocumab decreased lesion size, lipid accumulation, and inflammation in atherosclerotic plaques from ApoE^−^/^−^ mice. (**A**) Representative images of the maximal atherosclerotic lesion at the aortic arch after staining with ORO or after incubation with anti-CD68 or -CD80 antibodies. Quantifications are expressed as lesion area and percentage of lipid accumulation (ORO-staining) or CD68^+^ and CD80^+^ macrophages in total intimal plaque area. Scale bar, 100 µm. (**B**) Representative immunohistochemical staining of TLR4, NLRP3, and caspase-1 at maximal atherosclerotic lesions. Quantifications are expressed as a percentage of the total intimal plaque area. Triangles are arrow heads, as examples of positive staining. * *p* < 0.05 and ** *p* < 0.01 vs. ApoE^−^/^−^ mice, by unpaired two-tailed Student’s *t*-test. *N* = 5–9, per group. Scale bar, 100 µm.

**Figure 4:**
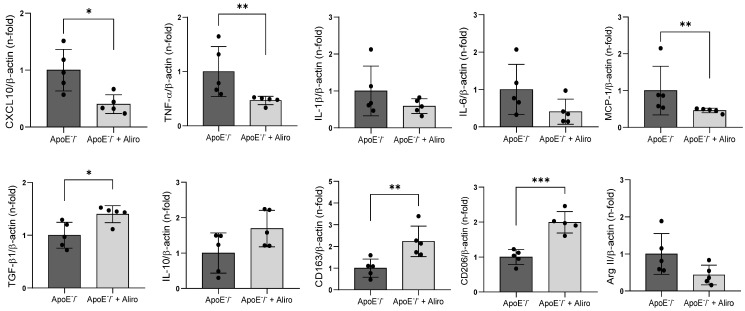
Alirocumab lessens the expression of proinflammatory cytokines while upregulating anti-inflammatory factors in ApoE^−^/^−^ mice. By qPCR, expression of proinflammatory *CXCL10*, *TNF-α*, *IL-1β*, *IL-6*, and *MCP-1* cytokines, and anti-inflammatory *TGF-β1* and *IL-10* in descending aorta from ApoE^−^/^−^ mice with/without alirocumab treatment. Specific markers for M2 (*CD163*, *CD206)* and M1 (*Arg II*) were also evaluated. * *p* < 0.05, ** *p* < 0.01, and *** *p* < 0.001 vs. ApoE^−^/^−^ mice, by unpaired two-tailed Student’s *t*-test or Mann–Whitney U test depending on data distribution. *N* = 5, per group.

We further investigated the potential anti-inflammatory effects of alirocumab in the atherosclerotic plaque. As previously described [14,25], ApoE^−^/^−^ mice exhibited prominent plaque lesions rich in lipid content at the aortic root (Figure 3A, top left). Importantly, alirocumab lessened plaque area (276 × 10^3^ µm^2^ vs. 655 × 10^3^ µm^2^; *p* < 0.05) and its lipid accumulation (3.1 ± 0.5% vs. 5.1 ± 2.3%; *p* < 0.01) (Figure 3A, top right). In addition, alirocumab diminished infiltration of CD68^+^-macrophages (2.3 ± 1.2% vs. 3.8 ± 0.8%; *p* < 0.05) and CD80^+^-M1 macrophages (7.1 ± 2.8% vs.19.4 ± 7.0%; *p* < 0.01) at the plaque lesion (Figure 3A, middle-bottom). Moreover, the TLR4 (2.3 ± 1.1% vs. 4.7 ± 1.1%; *p* < 0.01), NLRP3 (2.2 ± 1.2% vs. 4.3 ± 1.2%; *p* < 0.05), and caspase-1 (2.8 ± 1.1% vs. 5.3 ± 1.4%; *p* < 0.01) contents were also reduced after treatment (Figure 3B). Furthermore, in the descending aorta, the expression of major proinflammatory cytokines such as *CXCL10*, *TNF-α*, and *MCP-1* decreased after alirocumab, whereas anti-inflammatory cytokines (i.e., *TGF-β1*) and M2 macrophage membrane markers (i.e., *CD163* and *CD206*) were elevated (Figure 4).

### 3.4. PCSK9 Upregulated Inflammatory Factors in Cultured M1 Macrophages

**Figure 5:**
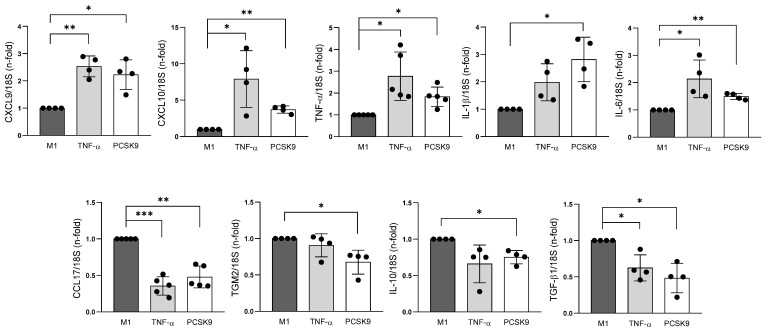
PCSK9 upregulated proinflammatory cytokines while decreasing anti-inflammatory factors in M1 macrophages. By qPCR, levels of proinflammatory *CXCL9*, *CXCL10*, *TNF-α*, *IL-1β*, and *IL-6* and anti-inflammatory *CCL17*, *TGM2*, *IL-10*, and *TGF-β1* cytokines in M1 macrophages stimulated with PCSK9. TNF-α was used as a positive control. * *p* < 0.05, ** *p* < 0.01, and *** *p* < 0.001 vs. unstimulated cells, by One-sample *t* test, control value set to 1. *N* = 4–5 independent biological replicates.

Next, we wondered whether PCSK9 might trigger direct proinflammatory actions on macrophages. Human THP-1 cells were polarized to M1 macrophages, as confirmed by elevation of the *CXCL9/CCL17* ratio [19] (Supplementary Figure S1C), and were incubated with recombinant PCSK9 for 24 h. Remarkedly, the expressions of proinflammatory cytokines, including *TNF-α* (1.8 ± 0.5-fold vs. unstimulated M1 cells; *p* < 0.05), *IL-1β* (2.8 ± 0.8-fold; *p* < 0.05), *IL-6* (1.5 ± 0.1-fold; *p* < 0.01), *CXCL9* (2.2 ± 0.5-fold; *p* < 0.05), and *CXCL10* (3.7 ± 0.5-fold; *p* < 0.05), were upregulated, in a similar manner than TNF-α (Figure 5, top). In contrast, PCSK9 downregulated anti-inflammatory cytokines such as *CCL17* (0.5 ± 0.1-fold vs. unstimulated M1 cells; *p* < 0.01), *TGM2* (0.7 ± 0.2-fold; *p* < 0.05), *IL-10* (0.8 ± 0.2-fold; *p* < 0.05), and *TGF-β1* (0.5 ± 0.2-fold; *p* < 0.01) (Figure 5, bottom).

**Figure 6:**
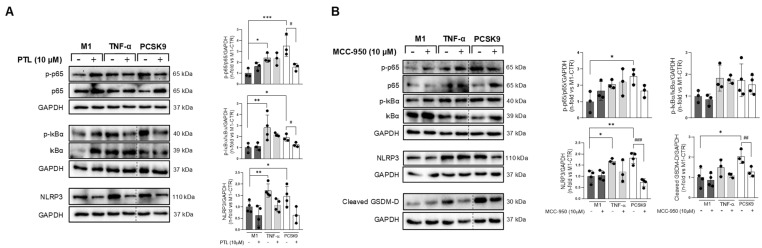
PCSK9 activated NFκB and NLRP3-inflammasome in M1 macrophages. (**A**) Phosphorylated and total p65 and IκBα were identified in PCSK9-stimulated M1 macrophages with/without pre-treatment of a NFκB inhibitor, parthenolide. Ratios of phosphorylated vs. non-phosphorylated were quantified. Bottom, NLRP3 was also detected and quantified. (**B**) NLRP3 and cleaved gasdermin-D were revealed after PCSK9 stimulation in M1 macrophages, with/without pre-treatment with a NLRP3 inhibitor (MCC-950). Phosphorylated and total p65 and IκBα were also uncovered, and the ratios of both isoforms were quantified. TNF-α was used as a positive control. * *p* < 0.05, ** *p* < 0.01, and *** *p* < 0.001 vs. unstimulated cells, by one-way ANOVA followed by Dunnett’s post hoc test, and ^#^ *p* > 0.05, ^##^ *p* > 0.01, and ^###^ *p* > 0.001 vs. PCSK9, by one-way ANOVA with Tukey’s correction. We used at least three independent biological replicates. Original western blot images can be found in Supplementary Figure S5.

In this sense, we also evaluated M1-polarized macrophages for the activation of major proinflammatory transcription factors. PCSK9 induced translocation of the NFκB p65 subunit to the nucleus after 3 h of incubation, similarly to TNF-α (Supplementary Figure S2). In fact, pre-treatment with the pharmacological NFκB inhibitor, parthenolide, attenuated this effect (Figure 6A). Parthenolide also reduced IκBα phosphorylation, which anchors p65 to the cytosol, and the expression of a major component of the inflammasome complex, NLRP3 (Figure 6A). Indeed, preincubation with MCC-950, an inhibitor of inflammasome assembly, significantly lessened PCSK9-induced NLRP3 expression, but not p65 or the inhibitor of κB alpha (IkBα) (Figure 6B). Subsequently, MCC-950 also led to a reduction in gasdermin-D cleavage, a downstream effector of caspase-1-mediated NLRP3 activation. Therefore, PCSK9 could promote direct proinflammatory actions in M1 macrophages through activation of NFκB and NLRP3-inflammasome.

### 3.5. TLR4, as a Potential Receptor for Proinflammatory Actions of PCSK9 in M1 Macrophages

**Figure 7:**
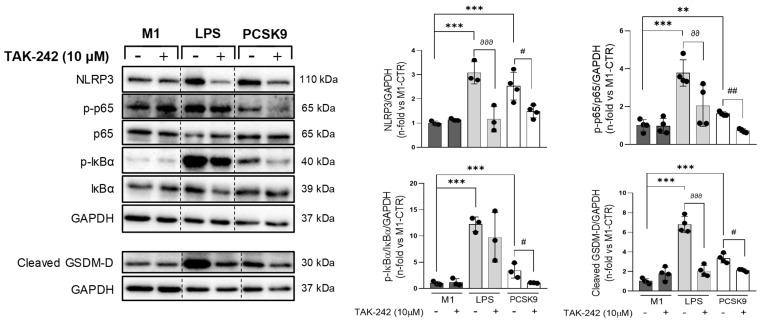
TLR4 inhibition dampened activation of the NFκB-NLRP3 axis upon PCSK9 stimulation. NLRP3 and cleaved gasdermin-D were identified after PCSK9 stimulation in M1 macrophages, with or without pre-treatment with a TLR4 inhibitor, TAK-242. Phosphorylated and total p65 and IκBα were also recognized, and the ratios of both isoforms were calculated. LPS was used as a positive control. ** *p* < 0.01 and *** *p* < 0.001 vs. unstimulated cells, by one-way ANOVA followed by Dunnett’s post hoc test. ^∂∂^ *p* < 0.01 and ^∂∂∂^ *p* < 0.001 vs. LPS, by one-way ANOVA with Tukey’s correction; and ^#^ *p* > 0.05 and ^##^ *p* < 0.01 vs. PCSK9, by one-way ANOVA with Tukey’s correction. We used at least three independent biological replicates. Original western blot images can be found in Supplementary Figure S5.

We then hypothesized that these direct PCSK9-proinflammatory actions might be mediated by pattern recognition receptors to translate danger signals from injured tissues to trigger inflammatory responses. Interestingly, in M1 macrophages stimulated with PCSK9, pre-treatment with a TLR4 antagonist, TAK-242, resulted in marked lessening of p65 and IκBα phosphorylation, as well as a decline in NLRP3 and cleaved gasdermin-D (Figure 7). Importantly, these effects were confirmed by TLR4 gene silencing in PCSK9-stimulated M1 macrophages (Supplementary Figure S3A). In turn, PCSK9 was able to increase TLR4 and its signaling adapter, MyD88 (1.9 ± 0.1 and 1.4 ± 0.1-fold; *p* < 0.01, respectively, Supplementary Figure S3B), suggesting that TLR4 may work as a critical downstream mediator of PCSK9 in M1 macrophages for proinflammatory outcomes.

### 3.6. Alirocumab Attenuated PCSK9-Induced Proinflammatory Responses in M1 Macrophages

Finally, we evaluated whether alirocumab directly counteracts PCSK9-induced inflammation in M1 macrophages. As expected, alirocumab significantly reduced the expression of *CXCL9* (2.3 ± 0.2 vs. 1.5 ± 0.1-fold in unstimulated cells; *p* < 0.001), *CXCL10* (1.5 ± 0.2 vs. 2.1 ± 0.3-fold; *p* < 0.05), *TNF-α* (1.0 ± 0.1 vs. 2.2 ± 0.5-fold; *p* < 0.001), *IL-1β* (0.9 ± 0.1 vs. 2.0 ± 0.4-fold; *p* < 0.01), and *IL-6* (1.6 ± 0.3 vs. 3.0 ± 1.4-fold; *p* < 0.05) promoted by PCSK9, while enhancing anti-inflammatory cytokines such as *CCL17* (0.9 ± 0.3 vs. 0.3 ± 0.1-fold; *p* < 0.01), *IL-10* (0.9 ± 0.4 vs. 0.4 ± 0.1-fold; *p* < 0.05), and *TGF-β1* (1.2 ± 0.8 vs. 0.8 ± 0.1-fold, *p* < 0.01) (Supplementary Figure S4A). Moreover, alirocumab significantly lessened phosphorylated IκBα and NLRP3 expression after PCSK9 exposure (Supplementary Figure S4B). In fact, alirocumab was able to diminish TLR4 transcripts, and perhaps Myd88, after PCSK9 stimulation (Supplementary Figure S3B). Altogether, PCSK9 could induce proinflammatory actions in M1 macrophages independently of LDL-C mitigation and via TLR4, NFκB, and NLRP3 signaling.

## 4. Discussion

In large-scale clinical trials like the ODYSSEY OUTCOMES, alirocumab demonstrated effective reduction of atherosclerotic plaques and cardiovascular events [26,27], but it may also reflect inhibition of PCSK9-driven anti-inflammatory effects. Other studies have reported that PCSK9 gene silencing in ApoE^−^/^−^ and LDLR^−^/^−^ mice attenuated aortic lesion development, macrophage infiltration, and the expression of proinflammatory cytokines, independently of LDL-C levels. In addition, PCSK9 overexpression in M0 macrophages was shown to enhance TNF-α and MCP-1 production through TLR4 and NFκB activation [10,23,28]. In this study, we provide further evidence that the PCSK9-neutralizing antibody, alirocumab, attenuates the progression of experimental atherosclerosis and modulates macrophage production and polarization. Moreover, in M1-polarized macrophages, PCSK9 activated the TLR4-NF-κB axis and the NLRP3 inflammasome, thereby amplifying proinflammatory responses.

The notion that PCSK9 may influence macrophage plasticity was previously suggested in experimental infarcted hearts. The PCSK9 gene silencing caused a reduction in M1 macrophage content and an increase in M2 phenotype, which ameliorated inflammatory infiltration and infarct size [29]. Also, PCSK9-knockout mice displayed a decreased inflammatory response to LPS [30]. Interestingly, PCSK9 overexpression in ApoE^−^/^−^ mice led to infiltration of proinflammatory Ly6C^high^ monocytes into atherosclerotic plaques, independently of plasma lipid levels [31]. In this sense, we observed that PCSK9 plasma levels were associated with other proinflammatory factors such as hsCRP and FGF-23 in patients with ACS, even after adjustment for LDL-C levels and previous use of statin or coronary disease. Although FGF-23 has been linked to adverse cardiovascular outcomes [32], no mechanistic conclusions can be drawn from our data. Moreover, in ApoE^−^/^−^ mice, alirocumab triggered a systemic anti-inflammatory phenotype characterized by a marked reduction in splenic M1 macrophages and elevation of reparative M2 cells, despite LDL-C levels being unaltered. Alirocumab also decreased plaque area, lipid accumulation, and overall macrophage infiltration, preferentially limiting the M1 subset. Although PCSK9 inhibitors have shown only modest effects on circulating inflammatory biomarkers in clinical studies, this does not preclude a relevant role of PCSK9 in vascular inflammation. In ApoE^−^/^−^ mice, the absence of apolipoprotein E severely impairs hepatic clearance of remnant lipoproteins, leading to persistent hypercholesterolemia. Consequently, PCSK9 inhibition does not reduce LDL-C levels in this model, allowing the evaluation of lipid-independent inflammatory actions of PCSK9. Therefore, the more pronounced anti-inflammatory effects should be interpreted as mechanistic evidence of PCSK9-driven inflammatory signaling rather than as a direct quantitative surrogate of circulating biomarker modulation in patients. In this line, clinical trials targeting inflammatory pathways, such as IL-1β blockade by canakinumab in the CANTOS trial or anti-leukocyte migration by colchicine in the LoDoCo2 study, have demonstrated that modulation of inflammation can reduce cardiovascular events [33]. In this sense, our findings suggest that alirocumab may modulate inflammatory responses associated with macrophage activation, which could reinforce the reduction in circulating mediators seen after canakinumab or colchicine. In consonance, evolocumab triggered lipid-independent effects, including enhancement of autophagy and reduction in oxidative stress to improve plaque size and composition [34]. Therefore, inhibition of PCSK9 by neutralizing antibodies might lead to additional non-lipid-mediated protective effects that could complement the anti-atherogenic actions of these drugs or other treatments (i.e., statins) [9]. Although the primary clinical role of alirocumab remains lipid-lowering, our data may help refine therapeutic concepts for patients with residual inflammatory risk, including those who remain vulnerable despite achieving lipid targets or who do not fully reach current lipid safety thresholds. Also, these effects may occur earlier than those elicited by a PCSK9 silencer such as inclisiran [35,36], though head-to-head studies will be determinative.

Concerning the molecular mechanisms, it is being postulated that polarization of macrophages within the atherosclerotic plaque is a key determinant of its progression and stability. Proinflammatory M1 macrophages, characterized by high TLR4 expression, dominate the microenvironment of advanced plaques [37]. Much of the existing literature has focused on demonstrating that PCSK9 can initiate the polarization of naïve macrophages towards this M1 phenotype [38]. Thus, our study specifically investigated PCSK9 effects on M1-activated macrophages and added new data on PCSK9 modulation of macrophage phenotype, immune signaling, and plaque inflammation [39]. In this line, we observed that PCSK9 exacerbated the M1 phenotype, increasing the CXCL9/CCL17 ratio and secretion of proinflammatory cytokines (IL-1β, IL-6, and TNF-α) via the TLR4-NFκB-NLRP3 pathway, similarly to TNF-α. This is consistent with previous studies on murine macrophages and endothelial cells, where PCSK9 overexpression stimulated IκBα phosphorylation and p65 translocation [40], while PCSK9 knockdown diminished TLR4 expression and NFκB activation in atherosclerotic plaque lesions [40]. Although our study was not aimed at exhaustively defining all upstream signaling intermediates, recent evidence suggests that PCSK9 may engage additional receptors and adaptor proteins upstream of TLR4. For instance, cyclase-associated protein-1 (CAP-1) has been identified as a high-affinity binding partner for PCSK9 in monocytes, acting upstream of TLR4 [41]. Thus, TLR4 can be an indispensable mediator to transduce PCSK9 signaling toward NFκB and downstream inflammasome activation. Activation of NFκB was previously suggested as an associated factor of PCSK9-stimulated monocytes [42,43], but the inflammasome could strengthen the proinflammatory signaling. Particularly, the NLRP3 inflammasome can be a critical innate immune sensor that influences atherosclerosis by detecting endogenous danger signals [44,45]. Its activation induces caspase-1-mediated IL-1β maturation and gasdermin-D cleavage, triggering pyroptosis and intensifying plaque inflammation [46]. Also, patients treated with PCSK9 inhibitors displayed lower NLRP3 and active caspase-1 expression within carotid plaques compared to those receiving other lipid-lowering therapies, even after adjustment for LDL-C levels [47]. Altogether, PCSK9-mediated stimulation of the TLR4-NFκB-NLRP3-Caspase-1 pathway may represent a key proinflammatory mechanism contributing to atherosclerosis progression.

## 5. Limitations of the Study

Our study has some limitations. Firstly, translating these findings to humans remains challenging since alirocumab is primarily a lipid-lowering therapy, and thus, its immunomodulatory effects should be considered additional or complementary rather than primary effects. Also, although our data clearly show increased gasdermin-D/Caspase-1 upon PCSK9 stimulation, additional studies are warranted to delineate the extent and regulation of pyroptotic signaling. Exploring additional ApoE^−^/^−^ mice models representing different stages of atherosclerosis, as well as testing multiple dose and timing regimens of alirocumab, could yield variable anti-inflammatory/-atherogenic responses. Finally, PCSK9 actions on other immune cells, such as neutrophil or monocyte-derived subtypes (i.e., M0 and M2 macrophages), may add interesting information on inflammatory responses after atherosclerosis.

## 6. Conclusions

In atherosclerosis, PCSK9 can be secreted concomitantly with proinflammatory cytokines and independently of LDL-C levels. Subsequently, PCSK9 may exacerbate the TLR4-NF-κB-NLRP3 signaling axis in M1 macrophages and reduce the M2 phenotype, thereby amplifying inflammatory responses and underscoring its deleterious, lipid-independent role in atherosclerosis. Notably, PCSK9 neutralization with alirocumab directly repressed these actions, suggesting a potential complementary strategy for attenuating atherosclerotic progression and, possibly, for mitigating other inflammation-driven cardiovascular diseases.

## Figures and Tables

**Table 1:** Alirocumab reduced plasma cytokines in ApoE^−^/^−^-Western diet-treated mice. Body weight, plasma lipid profile (total cholesterol, LDL-C, and triglycerides), and circulating proinflammatory cytokines (TNF-α and IL-6).

	ApoE^−^/^−^ (*n* = 9)	ApoE^−^/^−^ + Aliro (*n* = 7)	*p*-Value
Body weight (g)	31.4	31.0	0.62
Total cholesterol (mg/dL)	627.2 ± 113.3	653.2 ± 121.8	0.68
LDL-cholesterol (mg/dL)	403.9 ± 99.2	401.0 ± 101.7	0.95
Triglycerides (mg/dL)	226.7 ± 64.5	297.4 ± 106.4	0.15
TNF-α (pg/mL)	4.4 ± 1.0	3.2 ± 0.5	0.04
IL-6 (pg/mL)	4.4 ± 2.4	2.4 ± 0.6	0.04

## Data Availability

All data supporting this study are available within the Supplementary Material. Additional data related to the observational study are available upon reasonable request.

## Supplementary Materials

The following supporting information can be downloaded at: https://www.mdpi.com/article/10.3390/biom16030397/s1, Figure S1: (A) Gene silencing of TLR4 in M1 macrophages; (B) PCSK9 upregulation in ApoE treated mice; (C) CXCL9/CCL17 ratio in M1 macrophages. Figure S2: PCSK9 promoted nuclear translocation of the NFκB-p65 subunit in M1 macrophages; Figure S3: (A) Participation of TLR4 in the PCSK9-induced NFκB and NLRP3 activation, (B) Implication of a TLR4 mediator in PCSK9 inhibition; Figure S4: Alirocumab attenuated proinflammatory mediators (A) and genes (B) mediators induced by PCSK9 in M1 macrophages. Original western blots can be found at Supplementary Figure S5.

## Abbreviations

CCL17	C-C motif chemokine ligand 17
CXCL9	C-X-C motif chemokine ligand 9
CXCL10	C-X-C motif chemokine ligand 10
FGF-23	Fibroblast growth factor-23
hsCRP	High-sensitive C-reactive protein
IL-1β	Interleukin-1β
IL-6	Interleukin-6
IL-10	Interleukin-10
LDL-C	Low-density lipoprotein cholesterol
MCP-1	Monocyte chemoattractant protein-1
M1	Classically activated macrophage (proinflammatory phenotype)
M2	Alternatively activated macrophage (anti-inflammatory phenotype)
NFκB	Nuclear factor kappa-light-chain-enhancer of activated B cells
NLRP3	NOD-like receptor family pyrin domain-containing 3
PCSK9	Pro-protein convertase subtilisin/kexin type 9
TGF-β1	Transforming growth factor beta 1
TGM2	Transglutaminase 2
TLR	Toll-like receptor
