# Supplementary material for: Alirocumab Attenuated Plaque Inflammation and PCSK9-Induced Proinflammatory Signalling in M1 Macrophages Independently of Lipid Lowering
Source: Biomolecules. 2026 Mar 6;16(3):397. doi: 10.3390/biom16030397 (PMC13023508; doi:10.3390/biom16030397)
Supplement: Supplementary file 1 [file biomolecules-16-00397-s001.zip › biomolecules-4116719-Supplemental Figures S1-S4.pdf]

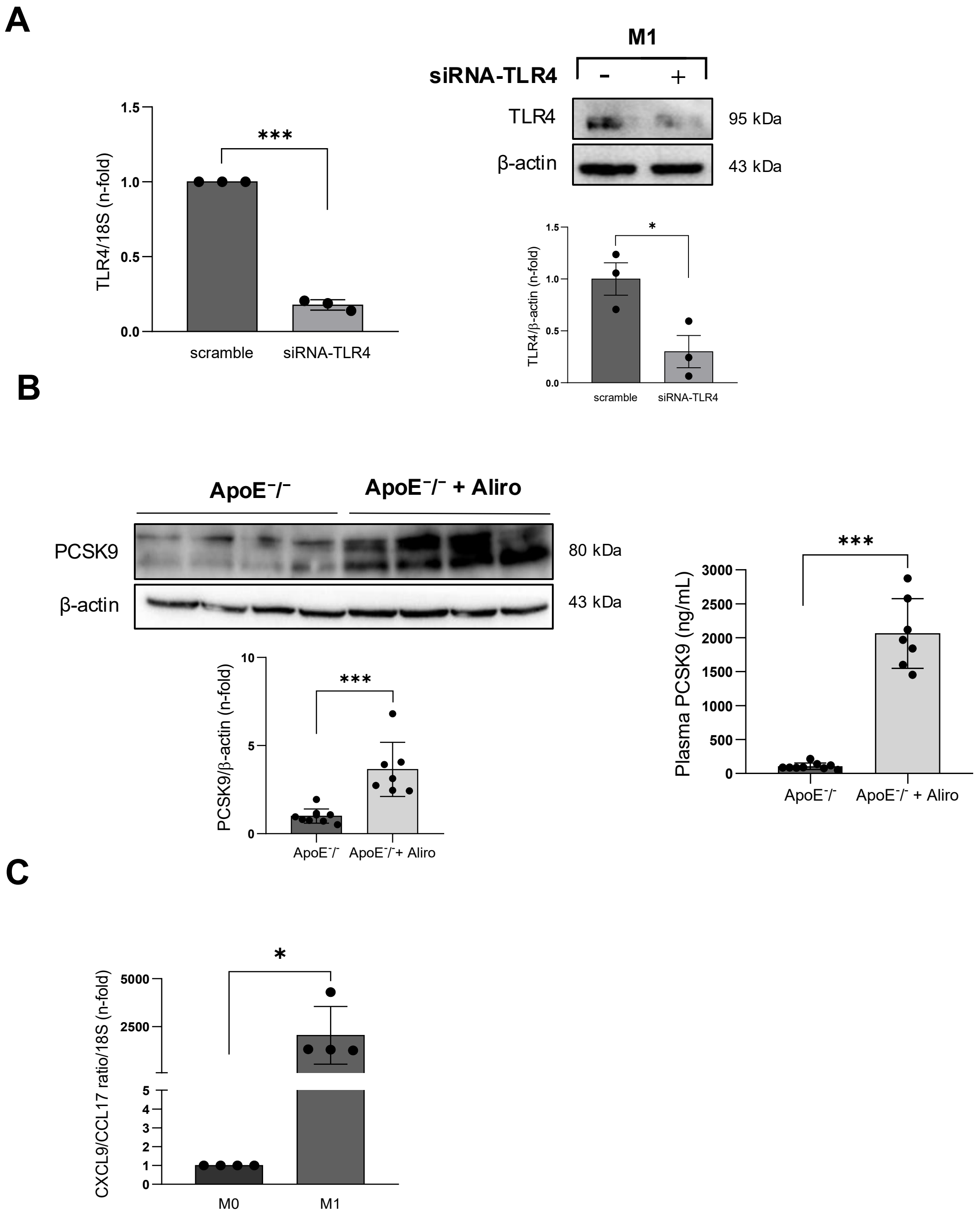

Supplementary Figure S1.

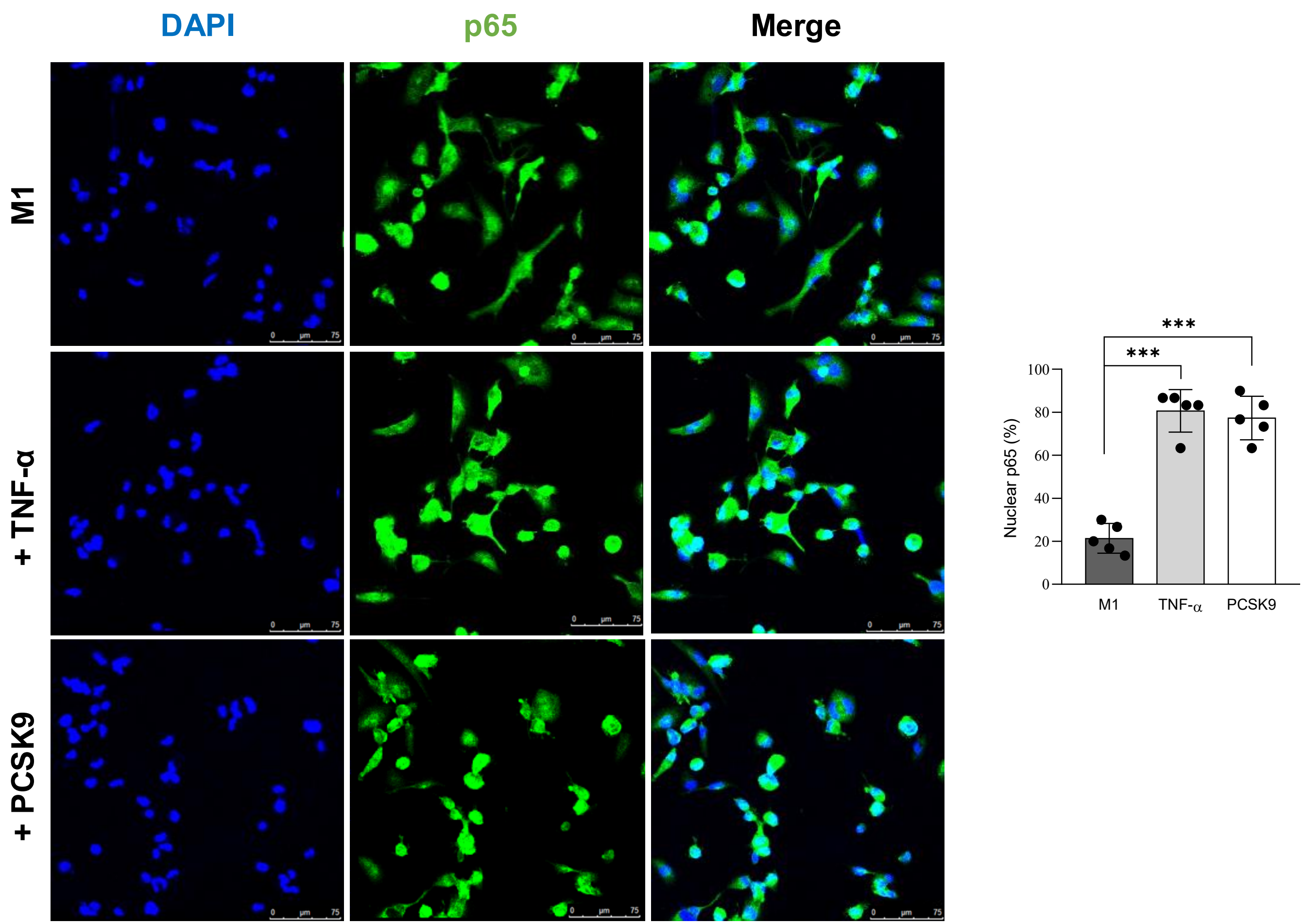

Supplementary Figure S2.

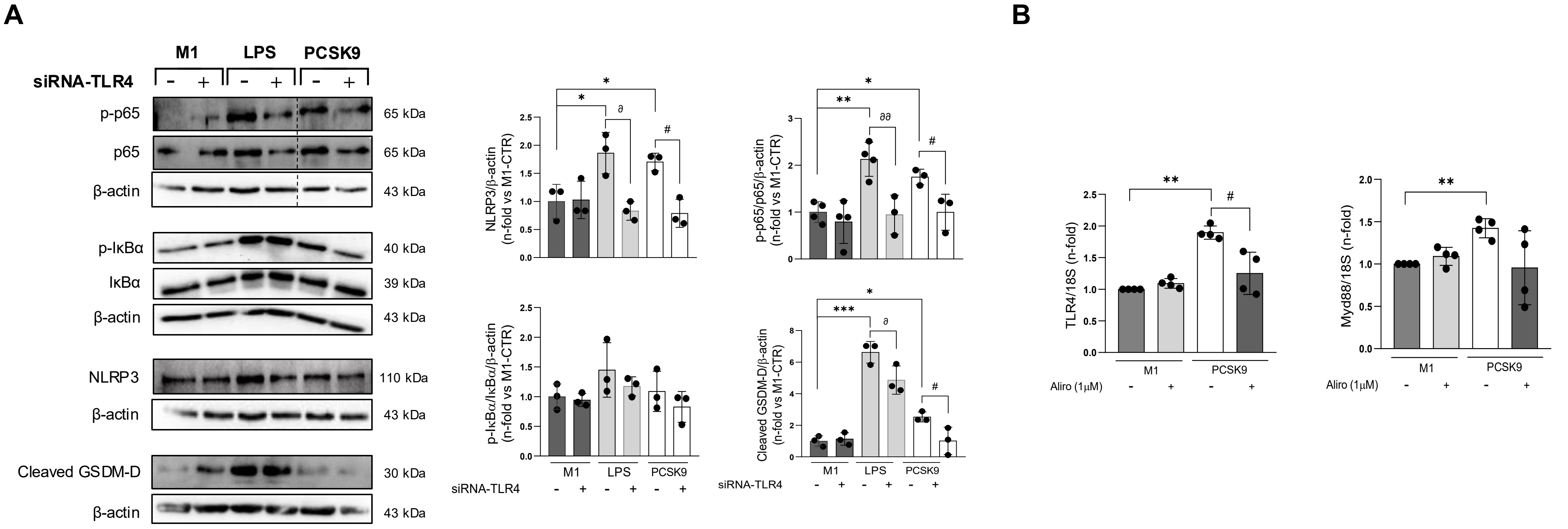

Supplementary Figure S3.

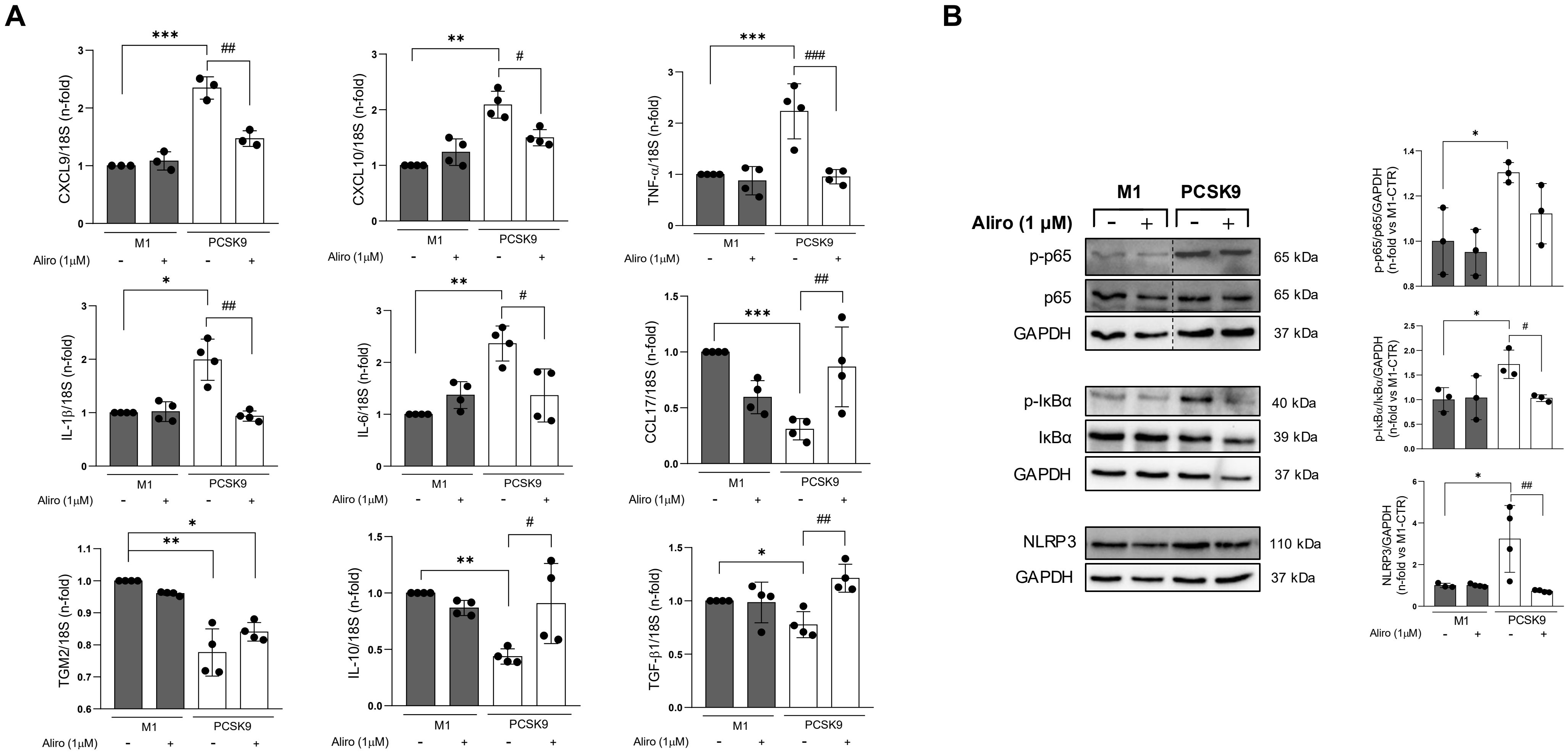

Supplementary Figure S4.

Figure S1: (A) Gene silencing of TLR4 in M1 macrophages. **(B)** PCSK9 upregulation in ApoE treated mice. **(C)** CXCL9/CCL17 ratio in M1 macrophages.

Figure S2: PCSK9 promoted nuclear translocation of the NFκB-p65 subunit in M1 macrophages

Figure S3: (A) Participation of TLR4 in the PCSK9-induced NFκB and NLRP3 activation. **(B)** Implication of a TLR4 mediator in PCSK9 inhibition

Figure S4: Alirocumab attenuated proinflammatory mediators **(A)** and genes **(B)** mediators induced by PCSK9 in M1 macrophages.
