# Supplementary material for: Alirocumab Attenuated Plaque Inflammation and PCSK9-Induced Proinflammatory Signalling in M1 Macrophages Independently of Lipid Lowering
Source: Biomolecules. 2026 Mar 6;16(3):397. doi: 10.3390/biom16030397 (PMC13023508; doi:10.3390/biom16030397)
Supplement: Supplementary file 1 [file biomolecules-16-00397-s001.zip › biomolecules-4116719-Supplemental Figure S5.pdf]

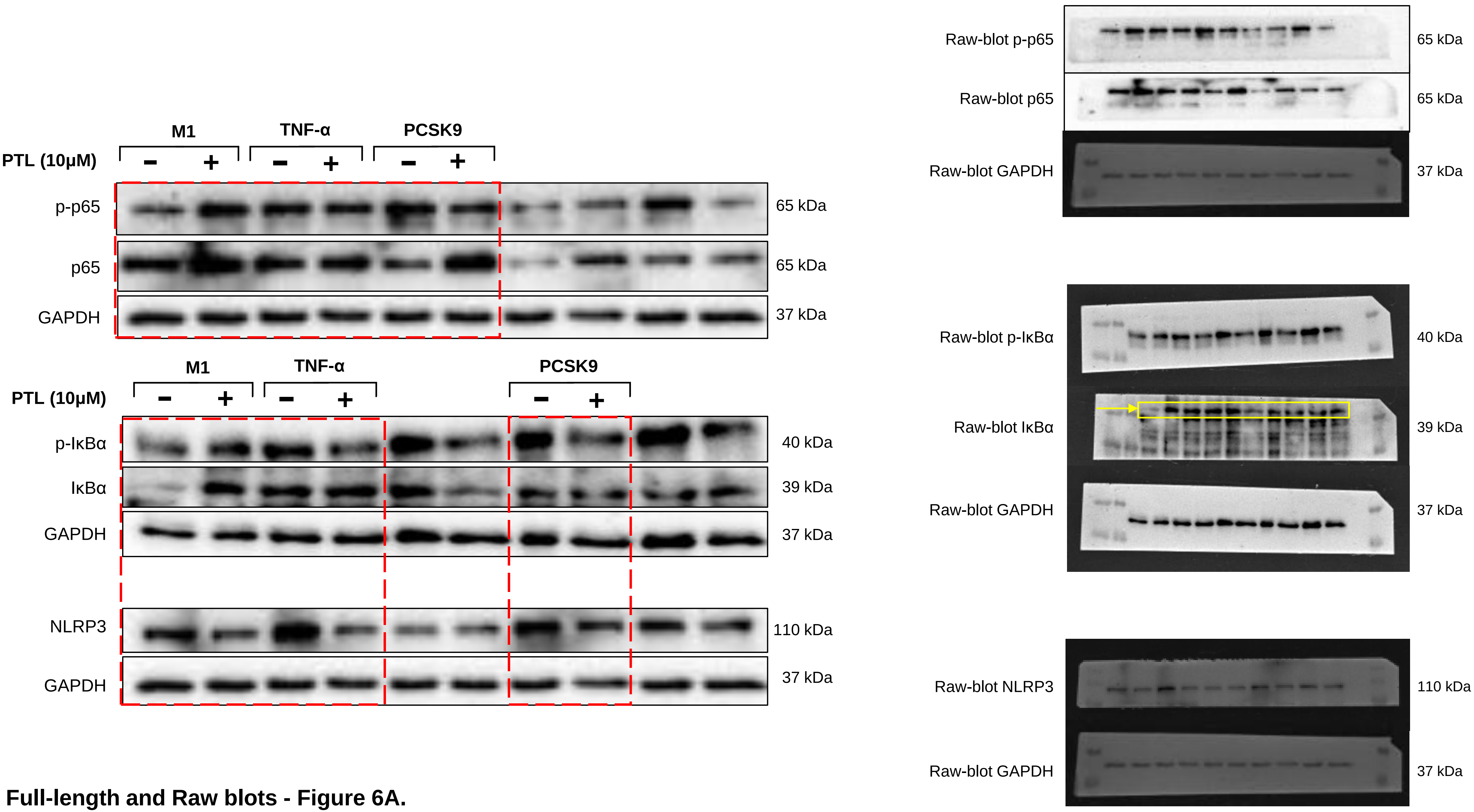

Full-length and Raw blots - Figure 6A.

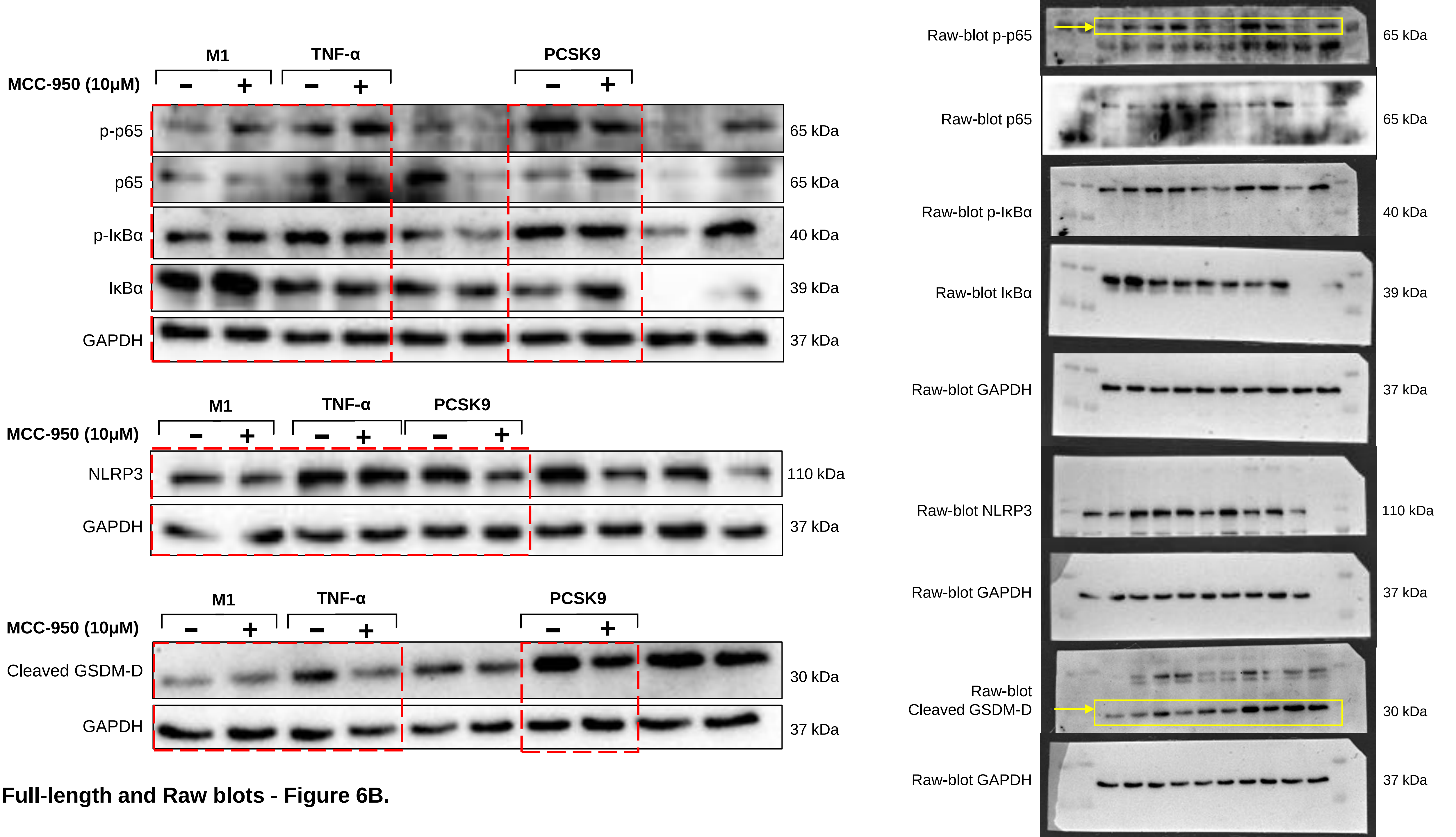

Full-length and Raw blots - Figure 6B.

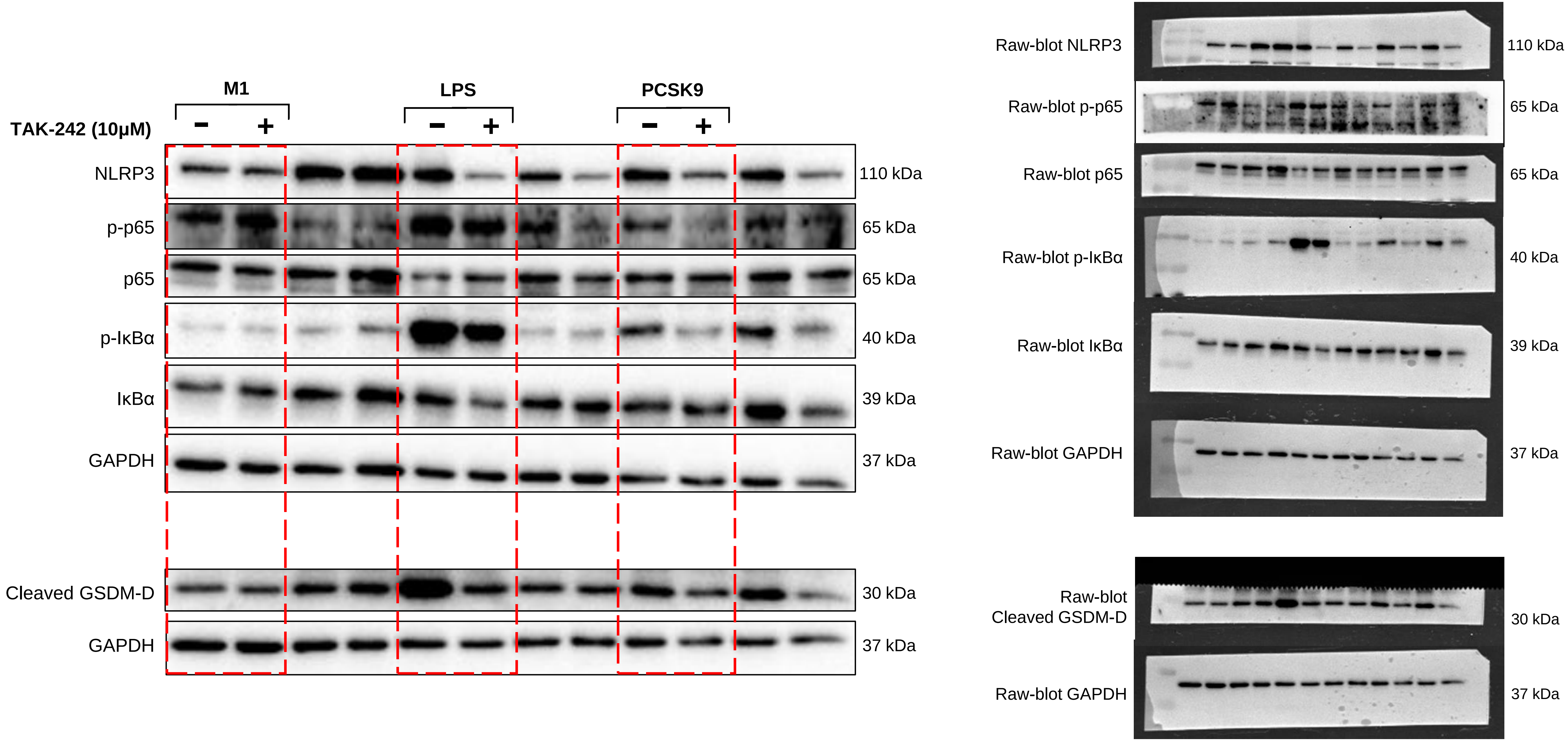

Full-length and Raw blots - Figure 7.

A

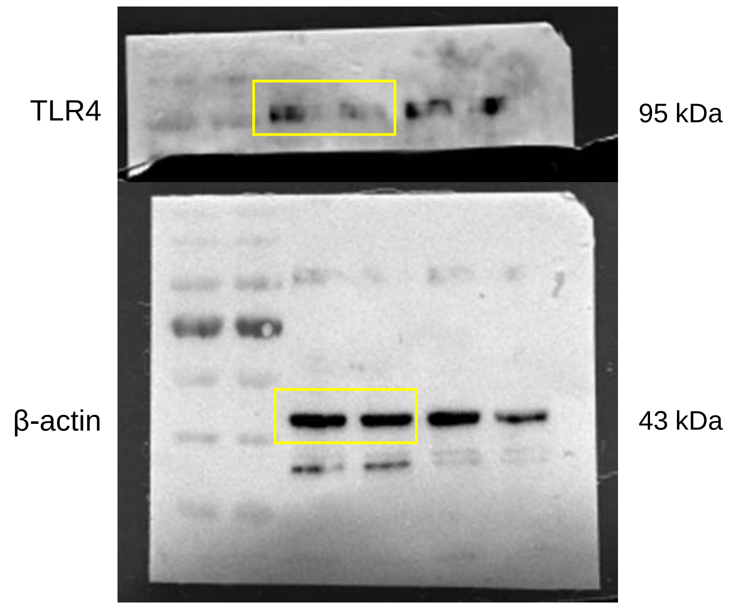

B

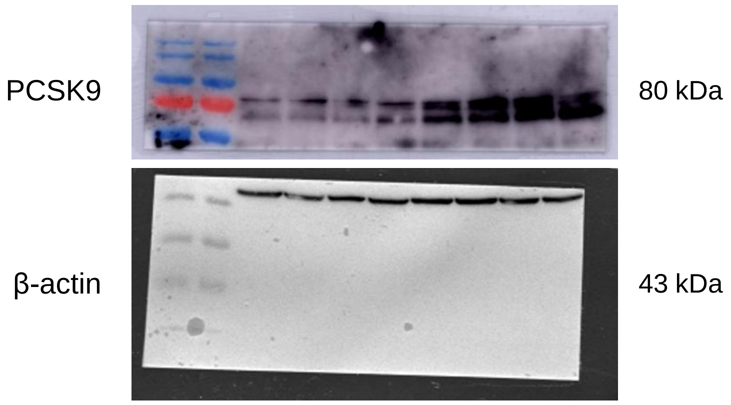

Full-length and Raw blots – Supplementary Figure S1.

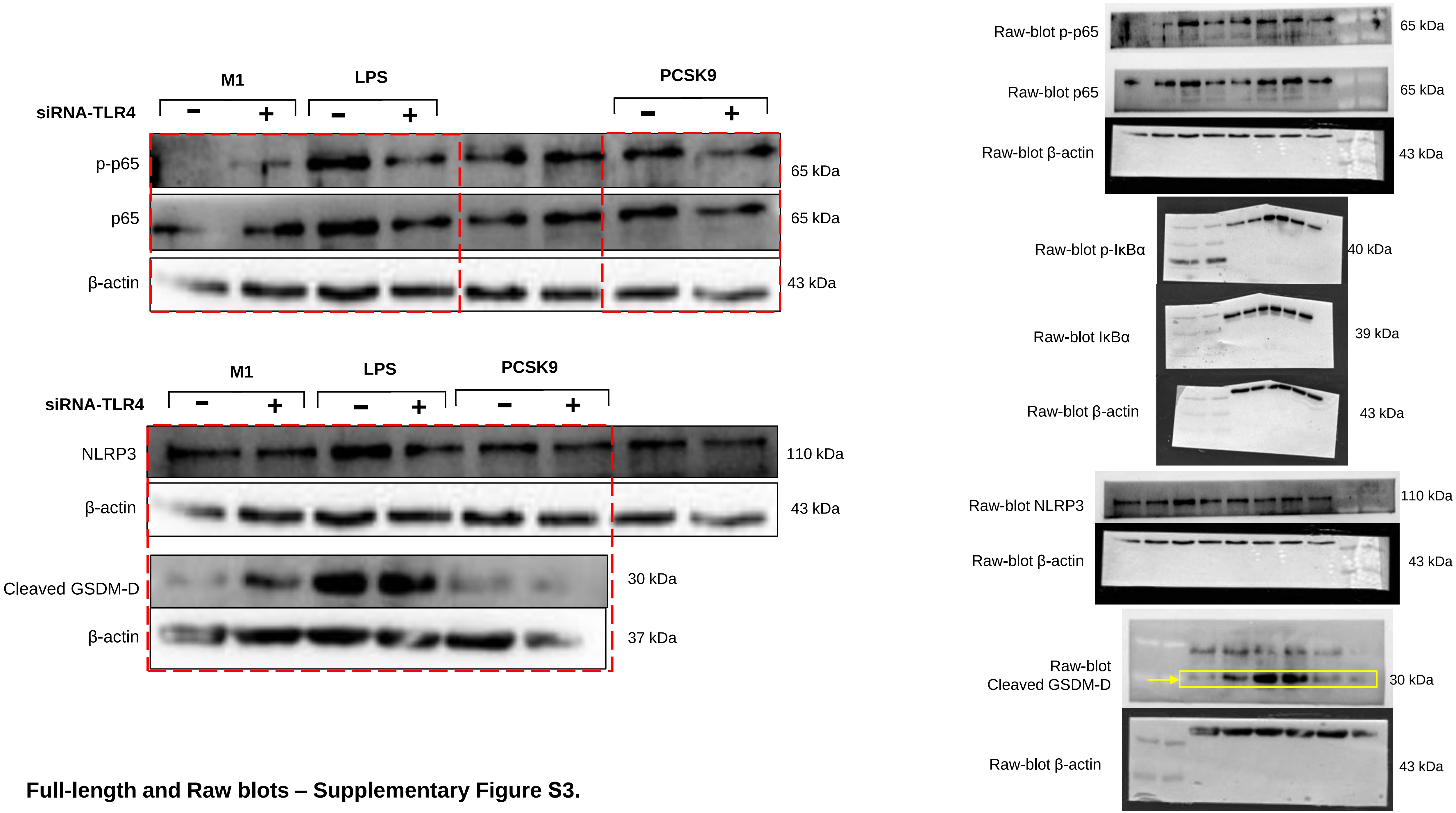

Full-length and Raw blots – Supplementary Figure S3.

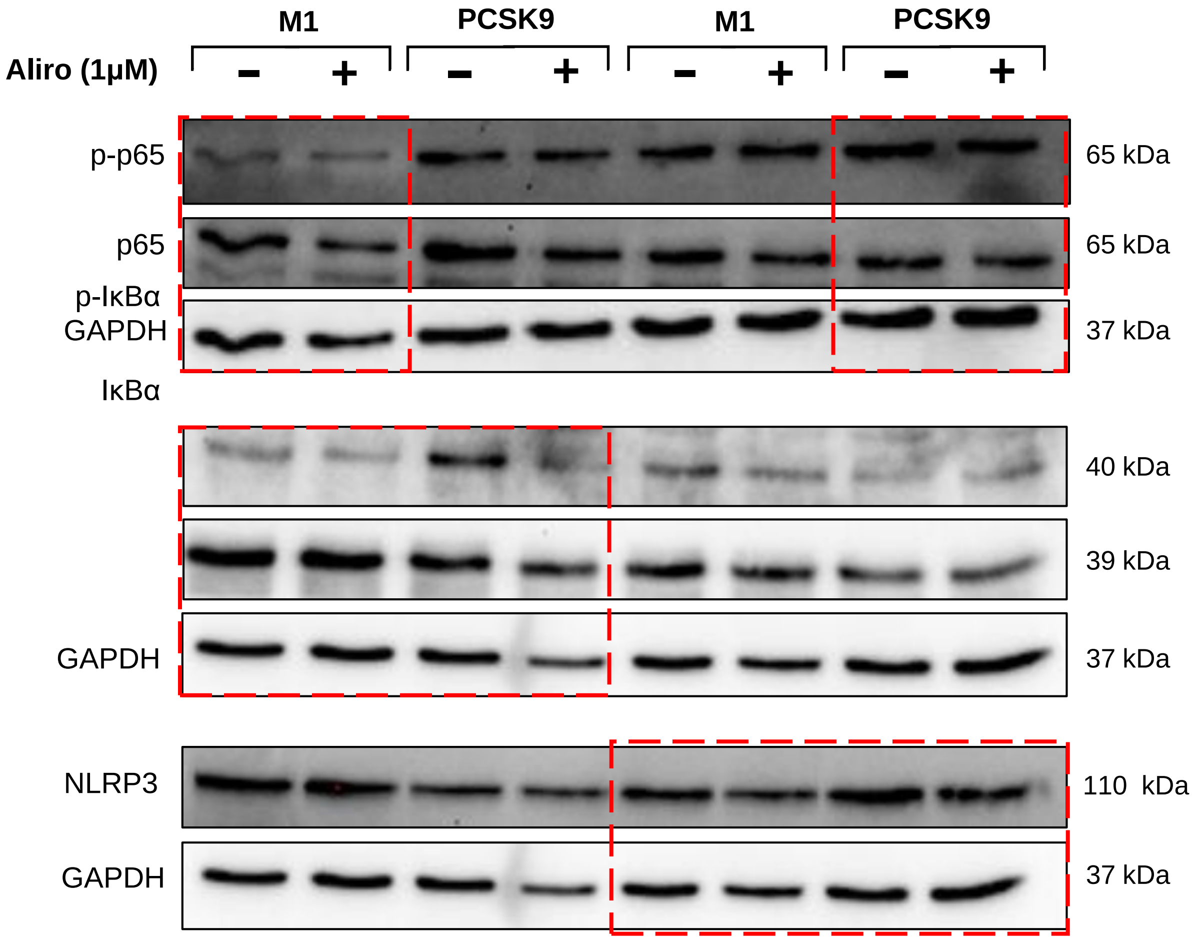

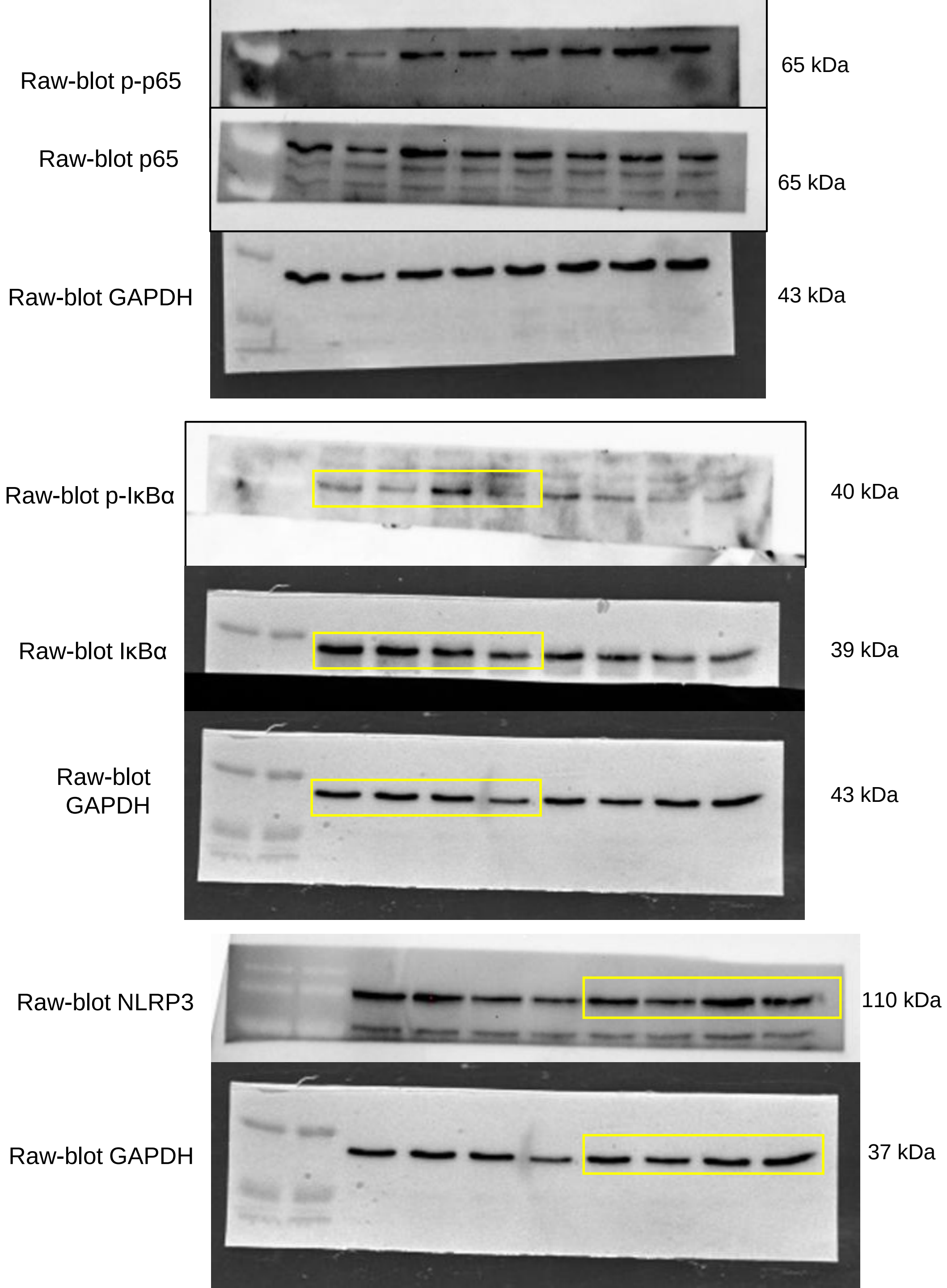

Full-length and Raw blots – Supplementary Figure S4.
